# YTHDC1-mediated VPS25 regulates cell cycle by targeting JAK-STAT signaling in human glioma cells

**DOI:** 10.1186/s12935-021-02304-0

**Published:** 2021-12-04

**Authors:** Xiaolong Zhu, Hui Yang, Mengying Zhang, Xingwei Wu, Lan Jiang, Xiaocen Liu, Kun Lv

**Affiliations:** 1 Central Laboratory, The First Affiliated Hospital of Wannan Medical College (Yijishan Hospital of Wannan Medical College), Wuhu, 241001 People’s Republic of China; 2 Key Laboratory of Non-Coding RNA Transformation Research of Anhui Higher Education Institutes (Wannan Medical College), Wuhu, 241001 People’s Republic of China; 3 Non-Coding RNA Research Center of Wannan Medical College, Wuhu, 241001 China; 4 Department of Nuclear Medicine, The First Affiliated Hospital of Wannan Medical College, Wuhu, 241001 Anhui People’s Republic of China

**Keywords:** YTHDC1, VPS25, Cell cycle, JAK–STAT, Glioma

## Abstract

**Background:**

Glioma is a common type of malignant brain tumor with a high mortality and relapse rate. The endosomal sorting complex required for transport (ESCRT) has been reported to be involved in tumorigenesis. However, the molecular mechanisms have not been clarified.

**Methods:**

Bioinformatics was used to screen the ESCRT subunits highly expressed in glioma tissues from The Cancer Genome Atlas (TCGA) and Gene Expression Omnibus (GEO) databases. The function of the ESCRT subunits in glioma cells was examined in vitro. Transcriptome sequencing analyzed the target genes and signaling pathways affected by the ESCRT subunit. Finally, the relationship between m^6^A (*N*^6^-methyladenosine) modification and high expression of the ESCRT subunit was studied.

**Results:**

VPS25 was upregulated in glioma tissues, which was correlated with poor prognosis in glioma patients. Furthermore, VPS25 knockdown inhibited the proliferation, blocked the cell cycle, and promoted apoptosis in glioma cells. Meanwhile, VPS25 induced a G0/G1 phase arrest of the cell cycle in glioma cells by directly mediating p21, CDK2, and cyclin E expression, and JAK-signal transducer and activator of transcription (STAT) activation. Finally, YTHDC1 inhibited glioma proliferation by reducing the expression of VPS25.

**Conclusion:**

These results suggest that VPS25 is a promising prognostic indicator and a potential therapeutic target for glioma.

**Supplementary Information:**

The online version contains supplementary material available at 10.1186/s12935-021-02304-0.

## Background

Glioma refers to a tumor originating from glial cells. It is the most common tumor in the central nervous system (CNS) and has the highest lethal rate [1]. According to the 2016 World Health Organization (WHO) classification, glioma can be classified into astrocytic tumors (astrocytoma, anaplastic astrocytoma, and glioblastoma), oligodendrogliomas, ependymomas, and mixed gliomas [2]. The 2015 annual report of the National Central Cancer Registry of China (NCCR) indicates that the morbidity and mortality of glioma patients are increasing year by year [3]. Glioma has a fast growth, easy invasion and metastasis, a high mortality, and a short average survival time. Thus, it has become a type of disease that causes great harm to human life and health [4]. At present, the traditional multi-mode therapy of surgical resection, radiotherapy, and chemotherapy has not significantly improved the prognosis of glioma patients [5]. Thus, exploring the underlying molecular mechanisms associated with glioma progression and metastasis may contribute to discovering more effective treatment methods and targets.

VPS25 is one subunit of the endosomal sorting complex required for transport (ESCRT) [6]. ESCRT is the molecular machinery in eukaryotic cells that recognizes the ubiquitinated cargo at the intraluminal vesicles (ILVs) and sorts them into late endosomes to form multivesicular bodies (MVBs). Its main function is to promote the degradation of ubiquitinated proteins, and it is also related to retrovirus budding, cell division, cell cycle, autophagy, fungi pH sensing, and transcriptional regulation [7–9]. The ESCRT system contains five complexes, termed ESCRT-0, I, II, and III, and a series of accessory proteins, such as VPS4 [10]. For example, the ESCRT-II complex comprises the VPS25, VPS22, and VPS36 subunits [11].

VPS25 regulates endosomal protein sorting by interacting with the CHMP6 subunit in the ESCRT-III complex, and the inhibition of this interaction prevents cell division and leads to cell death [12]. Studies in *Drosophila* have found that VPS25 is an unconventional tumor suppressor that prevents the non-autonomous hyperproliferation of cells by modulating NOTCH signaling [13, 14]. VPS25 also regulates HIV production and release through pre- and post-transcriptional mechanisms or by interacting with other ESCRT subunits [15]. Previous studies have shown that multiple ESCRT subunits are involved in tumorigenesis [16]. For example, the TSG101, which is overexpressed in most tumors, is positively associated with tumor formation [17]. VPS4A functions as a tumor suppressor in human hepatoma cells by regulating the secretion and uptake of extracellular exosomal microRNAs [18]. Current research shows that the role of ESCRT in tumorigenesis is due to the loss of transmembrane protein homeostasis and the resulting consequences on signal transduction, and it could account for a defective MVB pathway and cell cytokinesis. In addition, ESCRT is also thought to affect tumorigenesis through autophagy and exosomes [8].

In this study, we screened the high expression subunits of the ESCRT complex in glioma tissue. We found that the expression of VPS25 was upregulated in glioma and associated with poor prognosis of glioma patients. VPS25 was also necessary for glioma cells' proliferation, cell cycle, and apoptosis. Furthermore, m^6^A methyltransferases (METTL3 and METTL14) and m^6^A reader (YTHDC1) were found to regulate VPS25 expression.

## Materials and methods

### Patient samples

This study was reviewed and approved by the Ethics Committee of the First Affiliated Hospital of Wannan Medical College. A written informed consent was obtained from all the patients and/or their legal guardians. There were 66 glioma tissues and 14 normal brain tissues that were collected from patients diagnosed pathologically who underwent surgical resection at the Department of Neurosurgery of the First Affiliated Hospital of Wannan Medical College. Tissue samples were preserved in liquid nitrogen and stored at − 80 °C until RNA and protein extraction.

### Gene expression profiling and survival analysis

The mRNA expression array data of the ESCRT complex were downloaded from the Gene Expression Omnibus (GEO) database (accession number is GSE4290), which had been published previously [19], and The Cancer Genome Atlas database (TCGA, https://portal.gdc.cancer.gov/).

We used Gene Expression Profiling Interactive Analysis 2 (GEPIA2, https://gepia2.cancer-pku.cn/) [20] to analyze the VPS25 expression data and overall survival in glioma based on the TCGA and GTEx (Genotype-Tissue Expression) projects.

### Cell lines and culture

The human glioma cell lines U87MG and U251 were purchased from American Type Culture Collection (ATCC). Glioma cells were cultured in Dulbecco's modified Eagle medium–high glucose (DMEM; Hyclone, GE Healthcare Life Sciences, UT, USA) containing 10% (v/v) fetal bovine serum (FBS; Gibco, Life Technologies, NY, USA). All the cell lines were cultured in a humidified incubator with 5% CO_2_ at 37 °C.

### Real-time qPCR analysis

TRIzol reagent (Ambion, Life Technologies, CA, USA) was used for total RNA extraction from the tissues or cells according to the manufacturer's protocol described previously [21]. The RevertAid First Strand cDNA Synthesis Kit (Thermo Scientific, Lithuania, USA) was used for RNA reverse transcription. Real-time quantitative PCR was performed according to the instructions of QuantiNova™ SYBR® Green PCR Kit (Qiagen, Hilden, Germany) using the Bio-Rad CXF96 PCR system (Bio-Rad, Hercules, CA, USA). The relative gene expression of *VPS25* was calculated using the 2^−ΔΔCt^ method. *GAPDH* served as an internal control. The primers were synthesized by Sangon Biotech (Shanghai, China). *VPS25*: F-5′-TGGTGCTCGCTGGTCCTGT C-3′, R-5′-GGACTTGCTCTTATCCAACCACTCG-3′. *METTL3*: F-5′-TCAGCATC GGAACCAGCAAAG-3, R-5′-TCCTGACTGACCTTCTTGCTC-3′. *METTL14*: F-5′-GTTGGAACATGGATAGCCGC-3, R-5′-CAATGCTGTCGGCACTTTCA-3′. *YTHDC1*: F-5′-AGGGATCCTGAAAGGAGGGC-3, R-5′-CACTGCTGCCAGTCTC ATGG-3′.

### siRNA, shRNA, and overexpression of plasmid transfection

The negative control siRNA (NC) and *VPS25* siRNA (siVPS25) were synthesized by RiboBio (Guangzhou, China). The sequence of siRNA targeting the *VPS25-1*, *VPS25-3*, *METTL3*, *METTL14* and *YTHDC1* was GCACAAGGCCGAGATCATC, GTCGATCCAGATTGTATTA, CAAGTATGTTCACTATGAA, GAAGACGCCTT CATCTATT and CAAGGAGTGTTATCTTAAT. Cells (15 × 10^4^ cells/well) were seeded in a 6-well plate and, after 24 h, were transfected with siRNA at 100 nM by riboFECT™ CP Transfection Kit (RiboBio) according to the manufacturer's instructions. For the stable silencing, shRNA lentivirus (lenti-sh-NC, lenti-sh-VPS25) was purchased from HANBIO (Shanghai, China). Lentiviruses were transfected into glioma cells for 48 h, and stable cell clones were selected for 1 week using puromycin (5 μg/ml). For overexpression, the cDNA encoding VPS25 was subcloned into the pTSB02-GFP-PURO vector and transfected into U251 cells according to the manufacturer's protocol of Lipofectamine 3000 (Invitrogen, Carlsbad, CA).

### Cell proliferation and cycle assay

For the cell proliferation assay, the xCELLigence RTCA DP (ACEA Biosciences, CA, USA) was used as described previously [22]. At 24 h after transfection, cells were seeded in a 16-well E-plate (ACEA Biosciences) at a density of 8000 cells per well. The cell index was monitored by the xCELLigence system (ACEA Biosciences) in real time. In addition, the growth curve of the cell was analyzed by Excel (Microsoft, Washington, USA) or GraphPad Prism 5 (La Jolla, California, USA).

For the colony formation assay, transfected cells were seeded in a 6-well plate at 2 × 10^3^ cells per well for 2 weeks. The cells were washed twice with PBS, fixed with 4% paraformaldehyde, and stained with 0.5% crystal violet. The colonies were photographed and analyzed statistically.

For the cell cycle assay, transfected cells were incubated for 48 h and fixed in 75% ethanol at 4 °C overnight. Then the cells were washed twice with PBS and incubated in RNase A/PI (propidium iodide) solution (100 μg/ml RNase A and 50 μg/ml PI) at 37 °C for 30 min in the dark. The DNA contents of stained cells were analyzed using a Beckman Coulter FC500MPL flow cytometer (CA, USA). The data were analyzed by FlowJo V10 software (FlowJo LLC, OR, USA).

### Cell apoptosis assay

Flow cytometry with Annexin V and PI labeling was used to determine cell apoptosis. The transfected cells were incubated for 48 h and washed with PBS twice. Then cells were resuspended in 300 μl of binding buffer containing 5 μl Annexin V-FITC and 10 μl PI. After incubating for 15 min at room temperature in the dark, the cells were analyzed by a Beckman Coulter FC500MPL.

Apoptosis was detected by One Step TUNEL Apoptosis Assay Kit for TUNEL assay (Beyotime, Shanghai, China) according to the manufacturer's instructions. After TUNEL staining, the glioma cells were stained using DAPI and observed using a High-Content Imaging System (ImageXpress Micro Confocal, Molecular Devices, USA). The number of apoptotic cells is presented as a percentage of the total cells counted.

### RNA sequencing analysis

U251 cells were transfected with NC (n = 2) or siVPS25 (n = 2) for 48 h. Then, total RNA was extracted using the mirVana miRNA Isolation Kit (Ambion) following the manufacturer's protocol. The transcriptome sequencing and analysis were conducted by OE Biotech Co., Ltd. (Shanghai, China). The libraries were sequenced on the Illumina sequencing platform (HiSeq™ 2500, Illumina, CA, USA). Significantly differentially expressed genes (DEGs) between NC and siVPS25 cells were identified based on fold change ≥ 1.5 and P-value ≤ 0.05 using the DEGseq functions. Functional pathway analysis of DEGs was performed using KEGG pathway enrichment analysis.

### Western blots

The protein expression of p-JAK1, 2, 3 (Rabbit mAb; CST), p-STAT1, 2, 3 (Rabbit mAb; CST), CDK2 (Rabbit Polyclonal; Proteintech), cyclin E (Rabbit Polyclonal; Proteintech), p21 (Rabbit Polyclonal; Proteintech), VPS25 (Rabbit Polyclonal; Proteintech), METTL3, METTL14 and YTHDC1 (Rabbit Polyclonal; Proteintech) was detected by western blot as described previously [23, 24]. Briefly, cells were lysed by Sample Buffer, Laemmli 2 × Concentrate (Sigma, MO, USA) via the manufacturer's protocol. Next, the proteins were isolated by 10% SDS–PAGE gel and transferred to nitrocellulose membranes. After immunoblotting with primary and secondary antibodies, the protein bands were incubated with enhanced chemiluminescence (ECL) and visualized using a Tanon 5200 Chemiluminescence Imaging System (Shanghai, China).

### RNA immunoprecipitation

For quantification of m^6^A-modified *VPS25* levels, RNA immunoprecipitation (RIP) experiments were performed. First, RNA was incubated with anti-m^6^A (Mouse monoclonal, Abcam, MA, USA) or anti-IgG (CST) antibody for immunoprecipitation according to the standard protocol of a Magna RIP RNA-Binding Protein Immunoprecipitation Kit (Millipore, MA, USA). After RIP, the enrichment of m^6^A containing *VPS25* was detected via RT-qPCR assays.

### RNA m^6^A modification quantification

RNA m^6^A modification was quantified as described previously [25, 26]. Briefly, 200 ng of sample RNA was incubated with a diluted capture antibody. The m^6^A content of total mRNA was detected by an m^6^A-RNA methylation quantification kit (ab185912; Abcam, UK), following the manufacturer’s protocol. A microplate reader measured the m^6^A levels at a wavelength of 450 nm.

### In vivo xenograft mice experiments

BALB/c nude mice (4–5 weeks old, 10 mice) were purchased from the Experimental Animal Center of Qinglongshan (Nanjing, China) and raised in pathogen-free mouse colonies. The U87MG cells (1 × 10^7^ cells in 0.1 ml PBS) were injected subcutaneously into BALB/c nude mice. Tumor width and length were recorded every 5 days. The following formula calculated the volume of the tumors: volume = (length × width^2^)/2. The tumors were weighed after the mice were sacrificed. The ethics committee of the First Affiliated Hospital of Wannan Medical College approved the animal experiment.

### Immunohistochemistry (IHC)

For IHC staining, the paraffin-embedded tissue sections were deparaffinized and re-hydrated. After antigen retrieval and blocking endogenous activity, the sections were incubated overnight at 4 °C with primary antibodies, followed by HRP-conjugated secondary antibody at 37 °C for 1 h. DAB chromogenic agent was used to detect the VPS25.

### Statistical analyses

Data were presented as the mean ± S.D. of three independent experiments. GraphPad Prism 5 (La Jolla) was used for all the statistical analyses. Student's *t*-test was performed to compare the data statistically. *P < 0.05, **P < 0.01, and ***P < 0.001 were statistically significant.

## Results

### Screening for highly expressed ESCRT subunits in glioma

To examine the function of the ESCRT complex in glioma tumorigenesis, the expression of main ESCRT subunits was screened from two databases: TCGA and GEO (Additional file 1: Fig. S1 and Additional file 2: Fig. S2). In the TCGA database, 10 subunits were significantly increased, and 3 subunits were significantly decreased in glioma tissues compared to normal brain tissues. In the GEO database, 10 subunits were significantly increased, and 9 subunits were significantly decreased. Combining the two databases, we noticed that *MVB12A*, *VPS25*, *CHMP2A*, and *IST1* were highly expressed in glioma (Table 1). We then analyzed the relationship between the expression of these four genes and the pathological grade of glioma. We found that a higher expression of *VPS25* and *CHMP2A* was more frequent in tissues with advanced tumor stage from GEO and TCGA databases (Additional file 3: Fig. S3A–C).

**Table 1:** Expression of ESCRT subunits in normal brain tissues and glioma tissues from TCGA and GEO database

Complex	Proteins	TCGA		GEO	
		Expression	p-value	Expression	p-value
ESCRT-0	STAM2	Up	*	Up	ns
ESCRT-I	MVB12A	Up	***	Up	***
	MVB12B	Down	ns	Down	*
	TSG101	Up	**	Down	ns
	VPS28	Up	*	Up	***
	VPS37B	Up	*	Up	**
	VPS37C	Down	ns	Down	*
	VPS37D	Up	ns	Up	***
ESCRT-II	VPS25	Up	***	Up	***
	VPS36	Down	***	Down	***
ESCRT-III	CHMP1A	Up	ns	Up	*
	CHMP1B	Down	***	Down	***
	CHMP2A	Up	***	Up	***
	CHMP2B	Down	ns	Down	*
	CHMP3	Down	*	Down	***
	CHMP4A	Up	*	Down	ns
	CHMP4B	Down	ns	Up	ns
	CHMP4C	Up	ns	Up	*
	CHMP5	Up	ns	Down	***
	CHMP6	Up	**	Up	***
	CHMP7	Up	ns	Down	ns
ESCRT-others	IST1	Up	***	Up	***
	VPS4A	Down	ns	Down	*
	VPS4B	Down	ns	Down	***

ns: no significant

*P < 0.05, **P < 0.01, ***P < 0.001

### VPS25 is upregulated in glioma

**Fig. 1:**
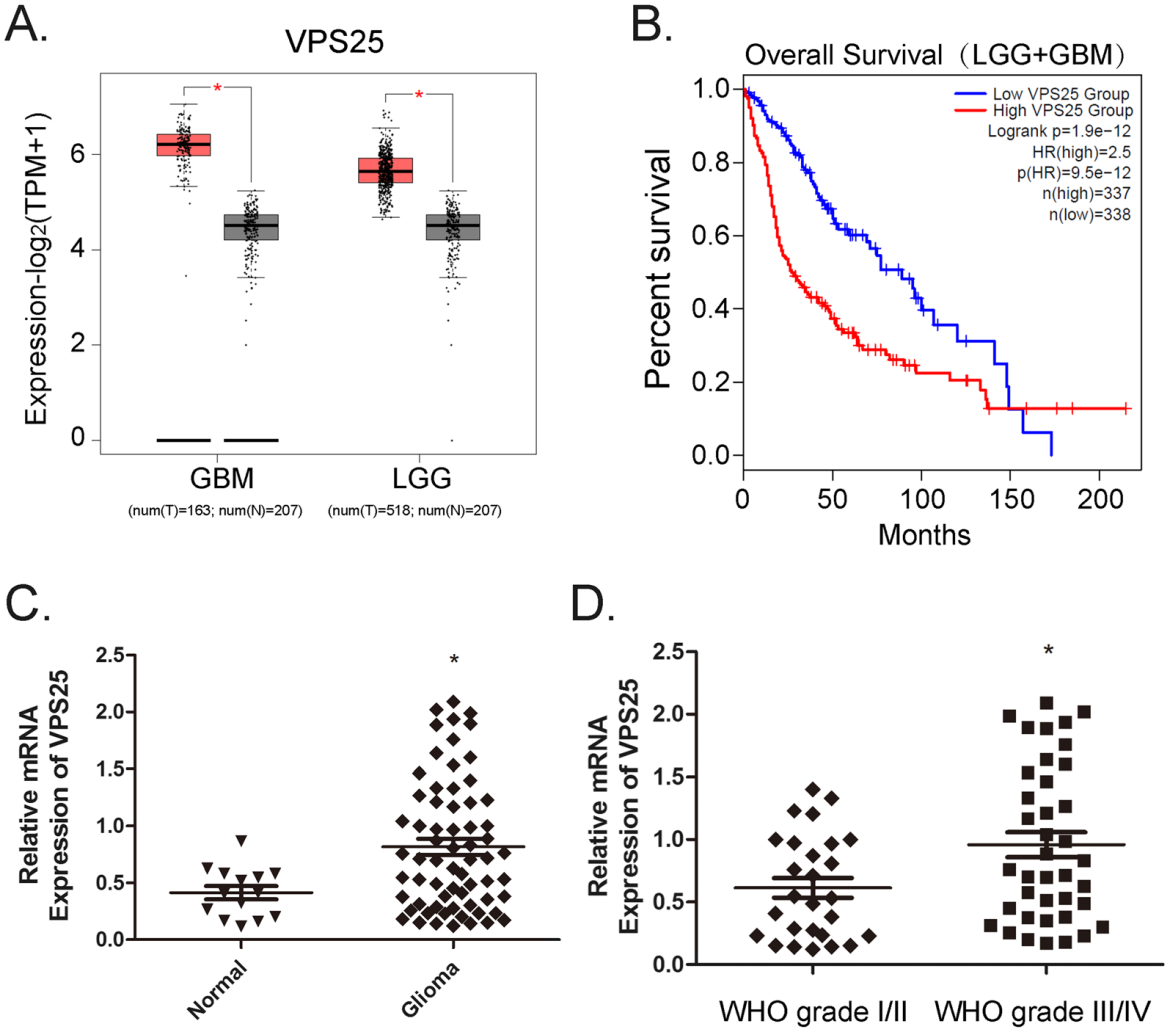
VPS25 is upregulated in glioma tissues. **A** The *VPS25* expression level in GBM or LGG was higher than in normal brain tissues based on data available from the GEPIA2 database. **B** The overall survival of glioma patients (LGG and GBM) with high (n = 337) and low (n = 338) levels of *VPS25* was plotted from GEPIA2. **C** *VPS25* in glioma was higher than in normal brain tissues. The relative expression levels of *VPS25* were determined using RT-qPCR in 66 glioma tissues and 14 normal brain tissues. **D** *VPS25* expression levels in WHO grade III/IV were higher than in WHO grade I/II. Data of *VPS25* expression in glioma tissues were from **A**. normal and N: non tumor samples; T: glioma; GBM: glioblastoma multiforme; LGG: lower grade glioma, * *P* < 0.05

The GEPIA2 database was used to confirm the expression and clinical particularities of VPS25. The expression of *VPS25* was significantly increased in glioma vs. controls (Fig. 1A). The glioma patients including both lower grade glioma (LGG) and GBM with high *VPS25* expression had a shorter survival time than those with low *VPS25* expression (Fig. 1B). However, high expression of *VPS25* was not associated with poor outcomes in LGG or GBM. (Additional file 3: Fig. S3D). We analysis the VPS25 expression in IDH-mutated and IDH-wildtype by Chinese Glioma Genome Atlas (CGGA) database (http://www.cgga.org.cn/index.jsp). In WHO II and III grade glioma, the VPS25 expression in IDH-mutated is higher than in IDH-wildtype (Additional file 3: Fig. S3E). After that, we determined the expression level of *VPS25* in normal brain (*n* = 14) and glioma tissues (*n* = 66) collected from Yijishan Hospital by RT-qPCR. Compared with the normal brain tissues, the *VPS25* expression was significantly increased in glioma tissues (Fig. 1C). The expression of the VPS25 protein in the GBM patient tissue is also higher than in normal tissues (Additional file 4: Figure S4). Further analysis showed that the expression of *VPS25* was increased in tissues with advanced tumor stage (Fig. 1D). Taken together, these results provided evidence that VPS25 upregulation may play an important role in the development of glioma.

### Function of VPS25 in glioma cell proliferation

**Fig. 2:**
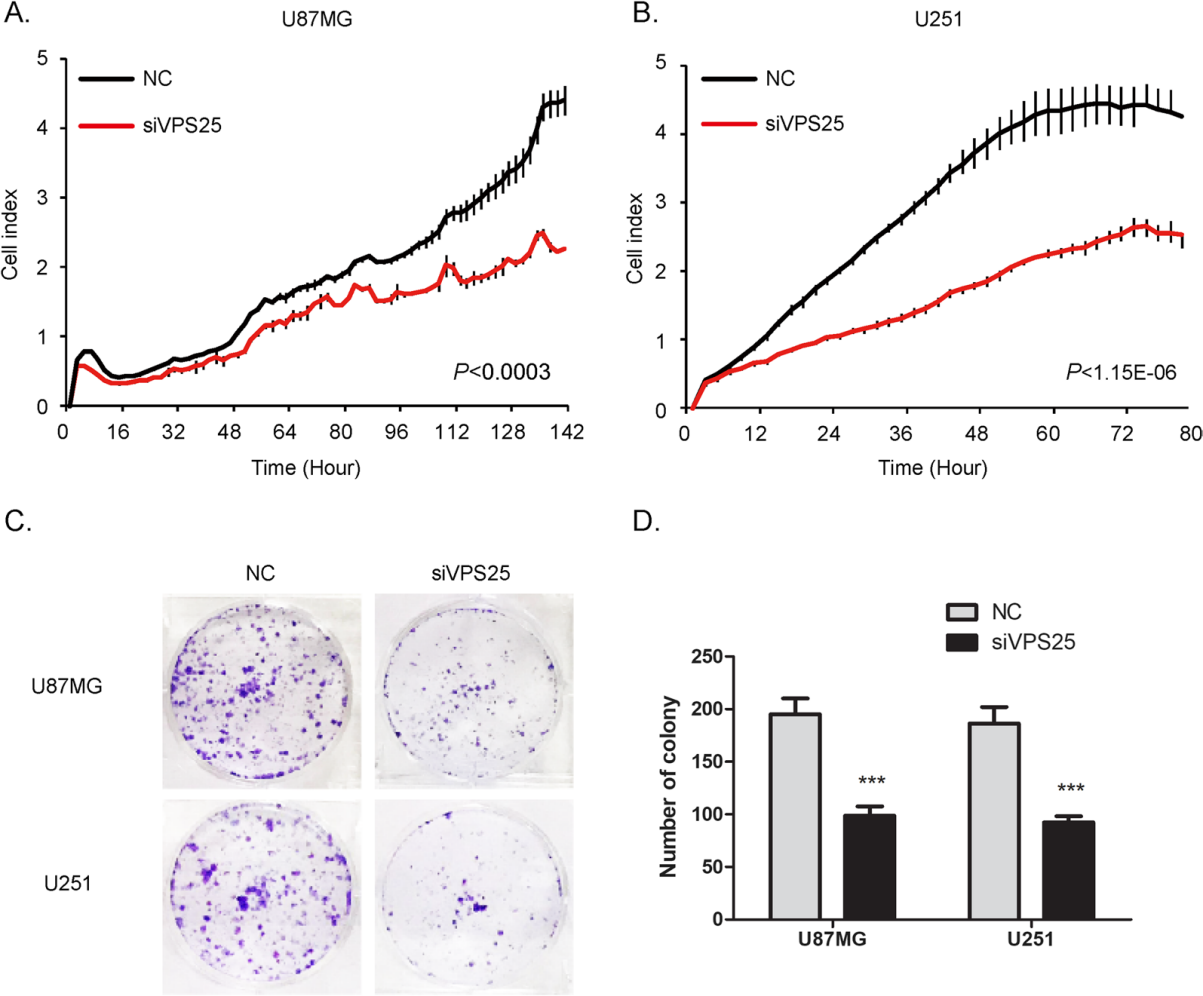
VPS25 regulates glioma cell proliferation. **A** RTCA xCELLigence assay showed that transfection of siVPS25 suppressed the proliferation of U87MG cells. **B** Transfection of siVPS25 suppressed proliferation of U251 cells. Experiments were performed and are presented as shown in **A**. **C** Transfection of siVPS25 suppressed the colony formation of U87MG and U251 cells. **D** Statistics of the colony number in **C**. Data are mean ± SD from three independent experiments. ***P < 0.001. NC: negative control; siVPS25: *VPS25* gene silencer

To verify the hypothesis that VPS25 functions as a candidate promoter in glioma, we examined the effect of VPS25 expression on cell proliferation and colony formation. RT-qPCR showed that *VPS25* expression was significantly increased in glioma cell lines (U87MG and U251), compared with VPS25 expression measured in normal brain tissues (Additional file 5: Fig. S5A). Then, *VPS25* siRNA was transfected to reduce the intracellular mRNA level of glioma. The RT-qPCR analysis revealed that transfection of glioma cells with siVPS25-3 significantly decreased the expression of *VPS25* by 95% in U87MG and 83% in U251 (Additional file 5: Fig. S5B and C). Here, we employed xCELLigence RTCA DP to evaluate the influence of VPS25 on cell proliferation. Compared with the NC, glioma cells transfected with the siVPS25 showed a remarkable inhibition of cell proliferation (Fig. 2A and B). Additionally, the colony formation assay was used to assess the long-term effects of VPS25 on cell proliferation. Fewer colonies were formed in glioma cells transfected with siVPS25 than in the NC (Fig. 2C and D). These data suggest that VPS25 positively regulates glioma cell proliferation in vitro.

### Function of VPS25 in glioma cell cycle and apoptosis

**Fig. 3:**
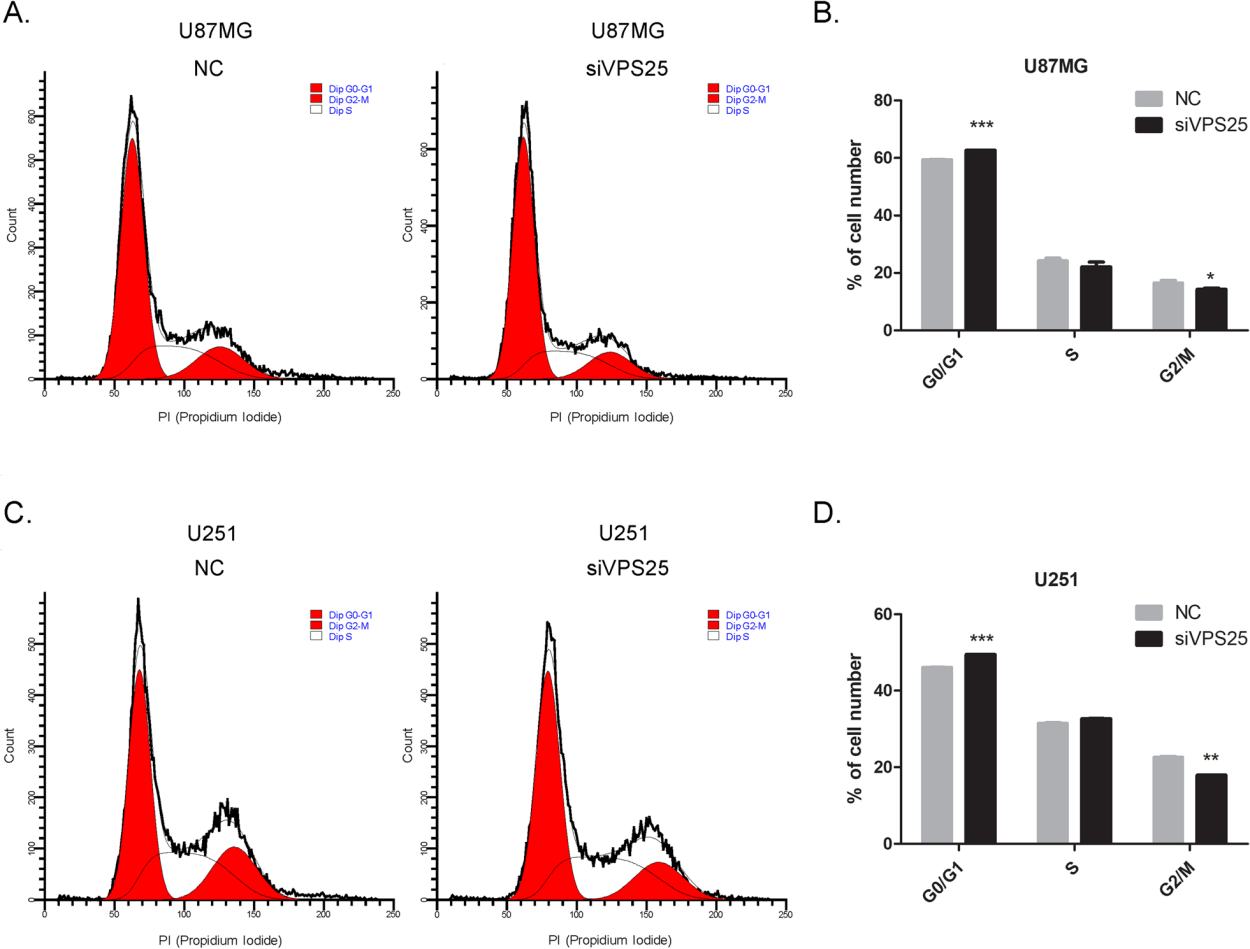
VPS25 regulates the glioma cell cycle. **A** The effect of VPS25 on the cell-cycle distribution of U87MG cells was monitored via flow cytometry. The *VPS25*-silenced U87MG cells were arrested at the G0/G1 phase of the cell cycle. **B** Histograms show the percentage (%) of cell populations at different stages of the cell cycle in **A**. **C** The *VPS25*-silenced U251 cells were arrested at the G0/G1 phase of the cell cycle. Experiments were performed and are presented as in **A**. **D** Histograms show the percentage (%) of cell populations at different stages of the cell cycle in **C**. Data are mean ± SD from three independent experiments. *P < 0.05, **P < 0.01, ***P < 0.001. NC: negative control; siVPS25: *VPS25* gene silencer

To confirm the function of VPS25 in cell proliferation, we investigated cell cycle distribution by flow cytometry. We found that the glioma cells under-expressing *VPS25* were arrested at the G0/G1 phase, with a corresponding decrease in the percentage of cells in the G2/M phase (Fig. 3). This result suggested that the *VPS25* knockdown (KD) inhibits glioma proliferation by blocking the cell cycle in the G0/G1 phase.

**Fig. 4:**
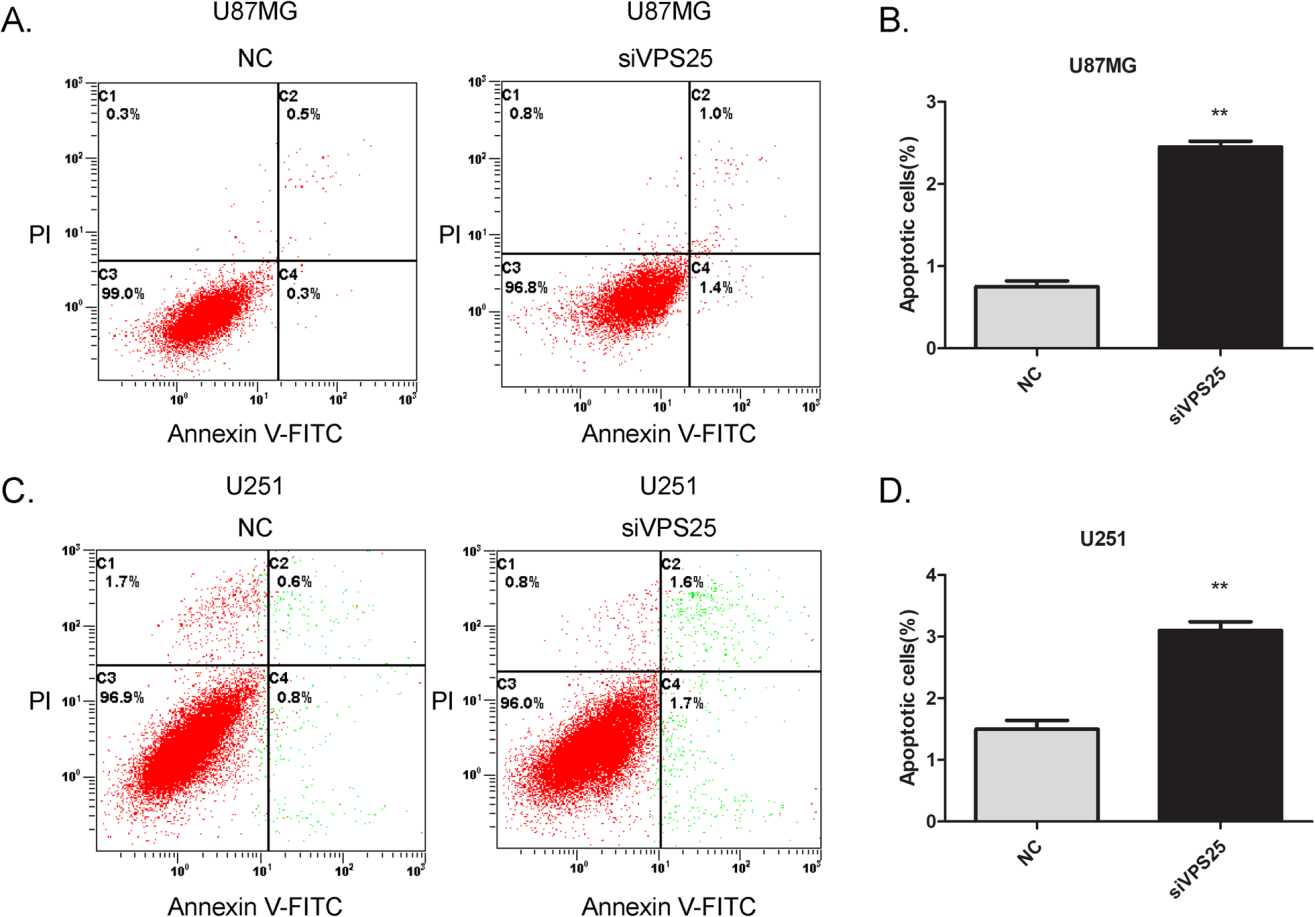
VPS25 regulates glioma cell apoptosis. **A** The Annexin V/PI analysis was used to evaluate the effect of VPS25 expression alteration on U87MG cell apoptosis. **B** Percentage of apoptotic cells in A. **C** The Annexin V/PI analysis was used to evaluate the effect of VPS25 expression alteration on U251 cell apoptosis. Experiments were performed and are presented as in **A**. **D** Percentage of apoptotic cells in **C**. Data are mean ± SD from three independent experiments. **P < 0.01, NC: negative control; siVPS25: *VPS25* gene silencer

To figure out the mechanism of VPS25-regulated cell proliferation, we used flow cytometry to detect glioma cell apoptosis. By double staining Annexin V-FITC/PI in glioma cells, the proportion of apoptotic cells transfected with siVPS25 was significantly higher than in the NC group (Fig. 4). Moreover, the TUNEL assay shown glioma cells treated with siVPS25 had a higher rate of apoptosis than cells treated with NC (Additional file 6: Fig. S6). These results indicate that VPS25 has a role in glioma cell apoptosis.

### Knockdown of *VPS25* represses glioma growth in vivo

**Fig. 5:**
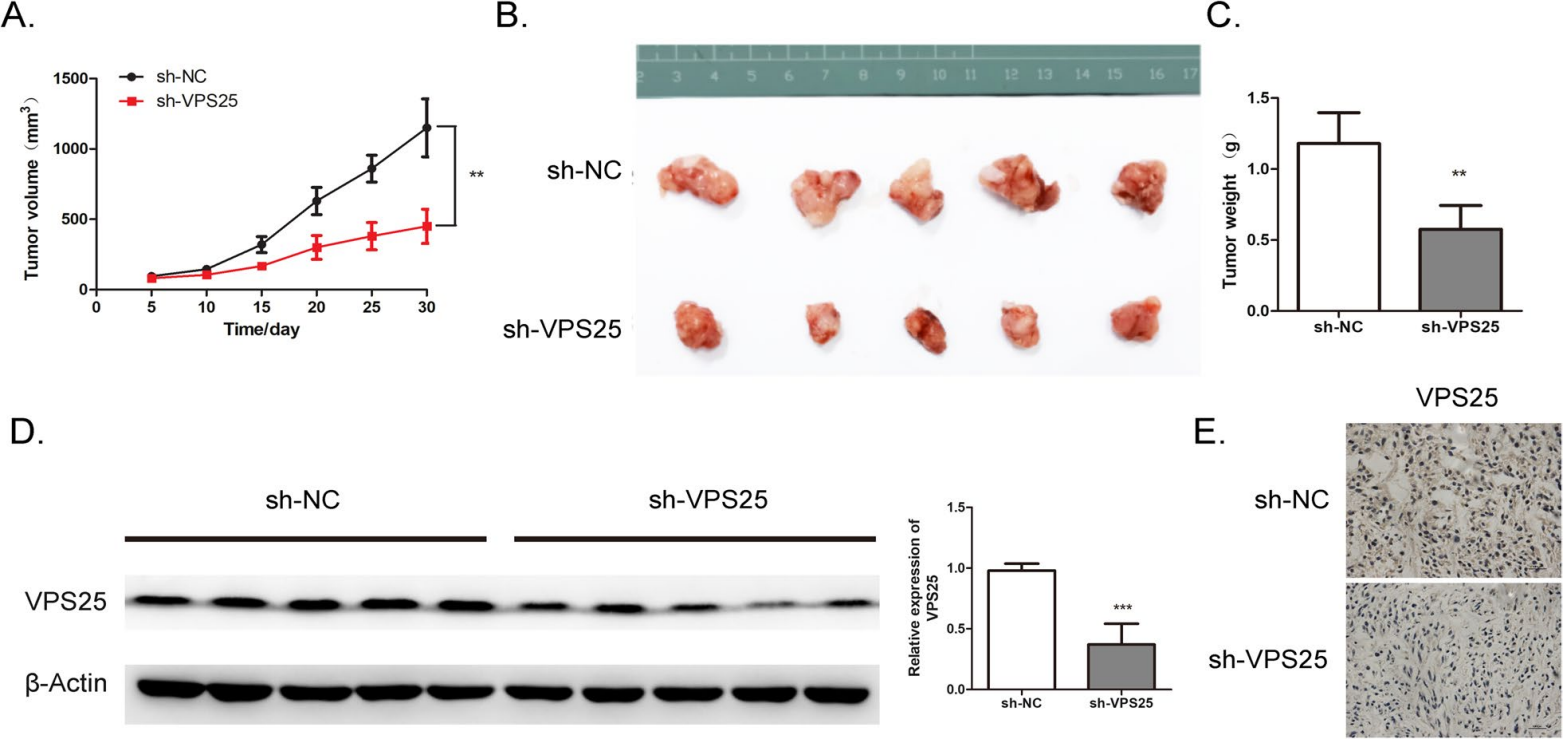
Knockdown of *VPS25* represses glioma growth in vivo. **A** A xenograft murine model was built using the U87MG cells transfected with *VPS25* stable knockdown (sh-VPS25) or control (sh-NC). The glioma volume curves were detected in sh-VPS25 and sh-NC groups for 30 days. **B** Tumors were collected from nude mice (sh-VPS25 and sh-NC) at 30 days. **C** Statistics of the weight of the tumors in **B**. **D** The VPS25 protein expression in sh-VPS25 and sh-NC groups was detected by western blot. **E** The VPS25 protein expression in sh-VPS25 and sh-NC groups was detected by immunohistochemistry. The results are shown as the mean ± SD. **P < 0.01, ***P < 0.001

To explore the role of VPS25 in glioma growth, stable *VPS25*-KD U251 cells were constructed using shRNA lentivirus (shVPS25). Next, a subcutaneous xenograft model was used. The growth of tumors (volume and weight) from *VPS25*-KD xenografts was inhibited compared with the tumors from the NC group (Fig. 5A–C). The VPS25 expression in the xenograft tumor was then examined by western blot and immunohistochemistry. As presented, VPS25 expression was downregulated in the *VPS25*-KD tumor group (Fig. 5D and E). In conclusion, these findings indicated that knockdown of VPS25 suppresses glioma growth in vivo.

### VPS25 regulates JAK-signal transducer and the activator of transcription (STAT) signaling pathway

**Fig. 6:**
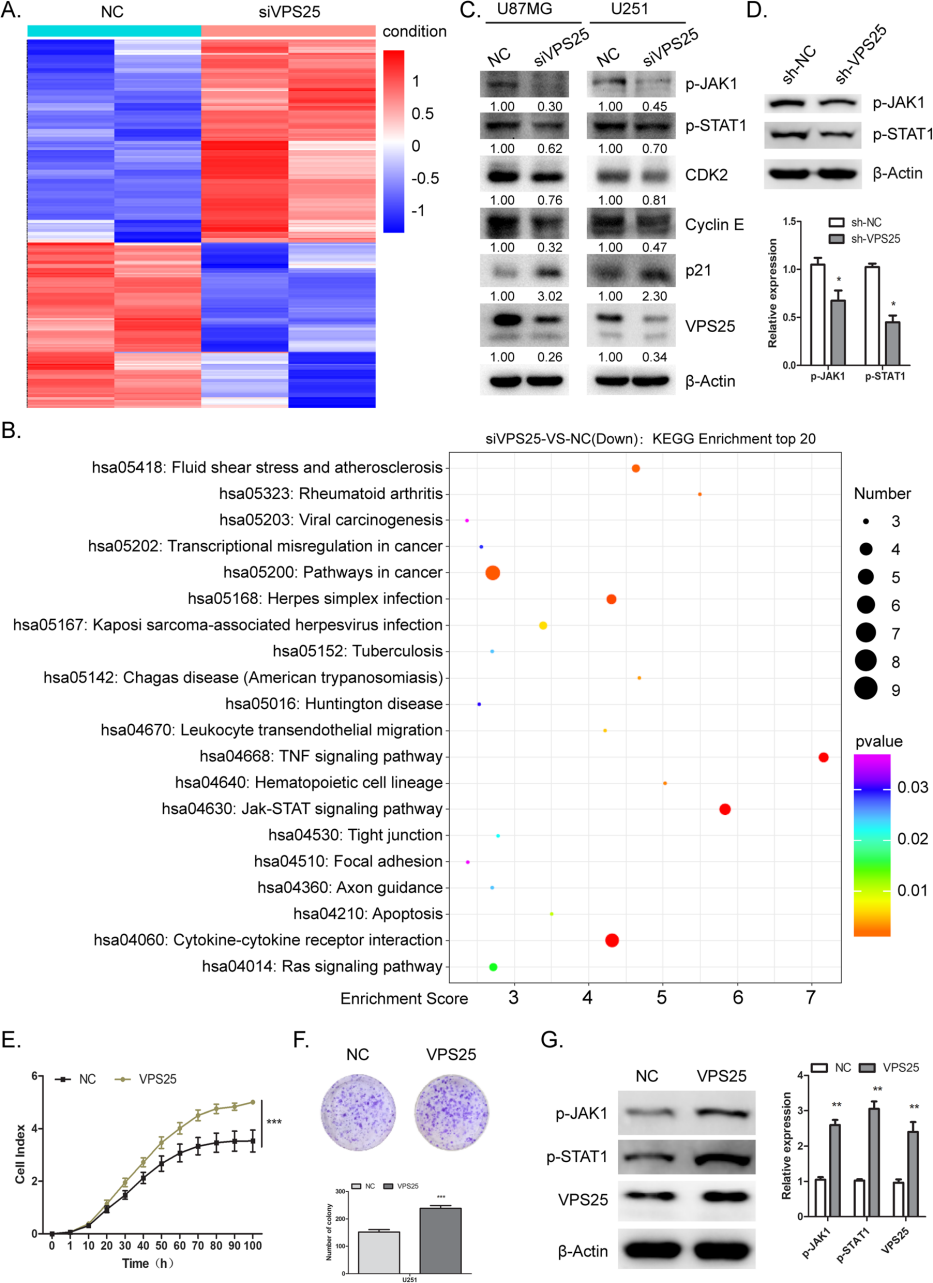
The genes regulated by VPS25 in glioma. **A** Heat map representing the DEGs regulated by *VPS25* KD. **B** KEGG pathway analysis showing the most enriched pathways of downregulated genes after *VPS25* KD. **C** Western blotting was used to determine the levels of downstream target proteins in *VPS25*-silenced U87MG and U251 cells. **D** Western blotting was used to determine the levels of p-JAK1 and p-STAT1 in *VPS25* KD tumor tissue in vivo. **E** RTCA xCELLigence assay showed that transfection of a VPS25-encoding plasmid promoted the proliferation of U251 cells. **F** Transfection of the VPS25-encoding plasmid promotes the colony formation of U251 cells. **G** Western blotting was used to determine the levels of p-JAK1 and p-STAT1 in VPS25 overexpressed U251 cells. β-Actin was used as a loading control. NC: negative control. siVPS25: *VPS25* gene silencer; VPS25: VPS25 overexpression plasmid. *P < 0.05, **P < 0.01, ***P < 0.001

To investigate the mechanism of VPS25 in glioma, we detected the DEG in *VPS25*-silenced U251 cells by RNA-seq. We identified 134 upregulated genes and 109 downregulated genes after *VPS25* KD (Fig. 6A and Additional file 7: Table S1). KEGG pathway enrichment analysis showed that the TNF signaling pathway, JAK-STAT signaling pathway, cytokine-cytokine receptor interaction, and herpes simplex infection were downregulated (Fig. 6B and Additional file 8: Table S2). The JAK-STAT signaling pathway plays a significant role in the cell cycle of various cancers [27]. We then checked the activity of JAK-STAT signaling by western blot in U87MG and U251 cells, as well as the *VPS25* KD tumor tissue in vivo. Phospho-JAK1 and phospho-STAT1 were decreased when *VPS25* was silenced (Fig. 6C and D). However, *VPS25* depletion did not affect other JAK and STAT proteins (Additional file 9: Fig. S7). After that, we detected the expression of various cell proliferation and cell cycle regulatory proteins known to be downstream targets of the JAK-STAT signaling pathway. We found that the cyclin-dependent kinase inhibitor p21 was significantly upregulated, whereas the cyclin E and cyclin-dependent kinase CDK2 were downregulated with reduced VPS25 (Fig. 6C). These results were consistent with our cell cycle data showing that *VPS25* KD led to a G0/G1 phase arrest. Then we employed another independent *VPS25* siRNA (siVPS25-1) to detect the function of VPS25 in glioma. *VPS25* knockdown inhibited the proliferation and blocked the cell cycle. Furthermore, *VPS25* knockdown reduced the expression of p-JAK1 and p-STAT1 (Additional file 10: Fig. S8).

To further elucidate whether VPS25 mediates proliferation through the JAK-STAT pathway, we overexpressed *VPS25* in the glioma cells. The results of the RTCA assay showed that *VPS25* overexpression promoted the proliferation of U251 cells (Fig. 6E). In addition, the colony-forming ability of U251 cells was increased due to *VPS25* overexpression (Fig. 6F). Furthermore, *VPS25* overexpression increased the protein expression of p-JAK1 and p-STAT1, noticeably in U251 (Fig. 6G).

### m^6^A modification is associated with *VPS25* expression in glioma cells

**Fig. 7:**
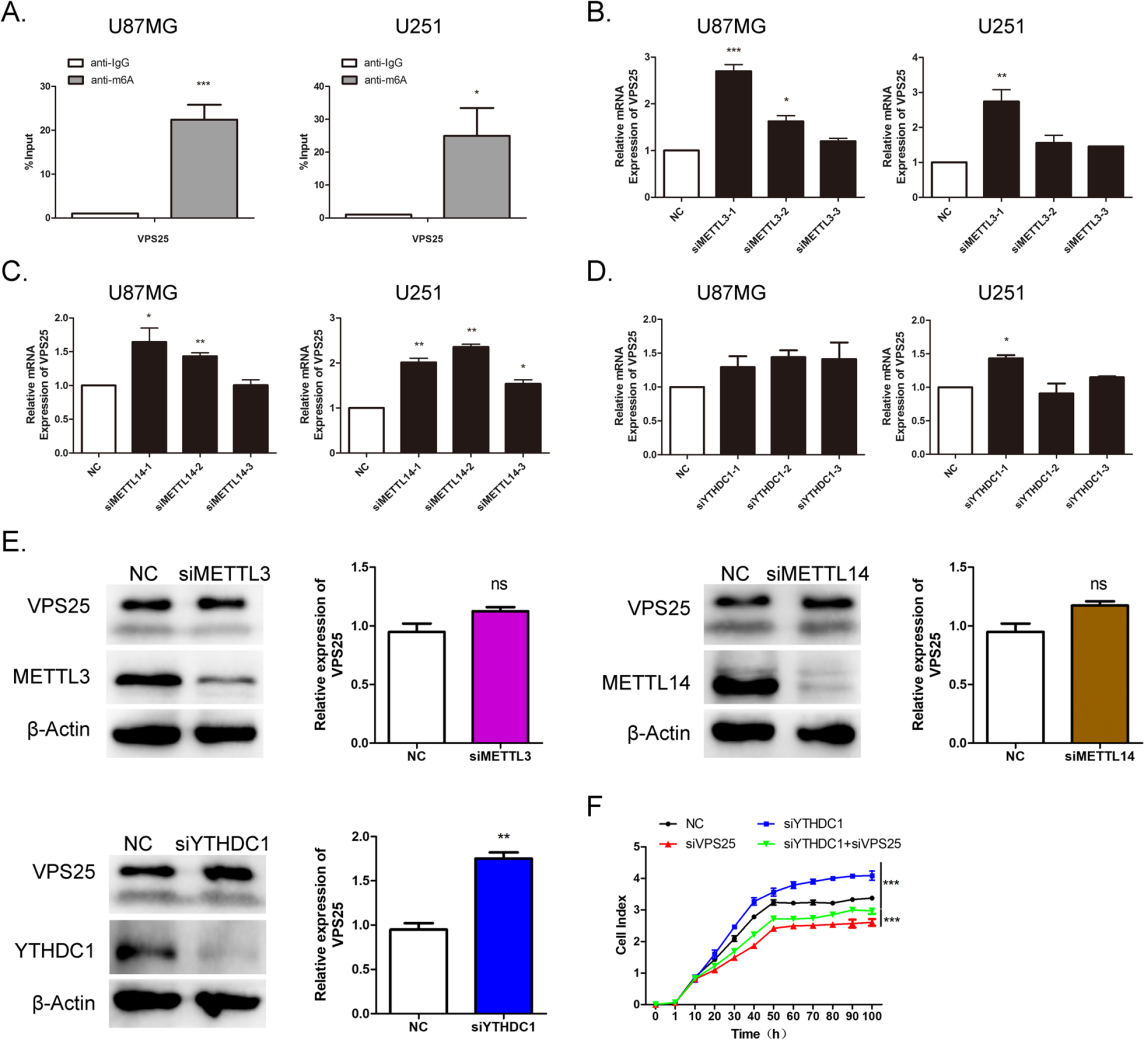
m^6^A modification is associated with *VPS25* expression in glioma cells. **A** m^6^A RIP-qPCR analysis showed that m^6^A is highly enriched within the *VPS25* transcript in U87MG and U251 cells. **B** The expression of *VPS25* was detected in U87MG and U251 cells with *METTL3* KD. **C** The expression of *VPS25* was detected in U87MG, and U251 cells with *METTL14* KD. **D** The expression of *VPS25* was detected in U87MG and U251 cells with *YTHDC1* KD. **E** Western blot was used to detect the *VPS25* expression in *METTL3*, *METTL14*, or *YTHDC1* KD cells. **F** U251 glioma cells were used to evaluate proliferation after being transfected with siVPS25 and siYTHDC1. *P < 0.05, **P < 0.01, ***P < 0.001. ns: no significant, NC: negative control. siMETTL3: *METTL3* gene silencer; siMETTL14: *METTL14* gene silencer; siYTHDC1: *YTHDC1* gene silencer; siVPS25: *VPS25* gene silencer

Recent studies in epigenetic mechanisms have shown that m^6^A modification in mRNAs plays critical roles in various cancers. Therefore, we investigated whether the upregulation of *VPS25* in glioma was associated with the m^6^A modification. According to the RMBase v2.0 database (http://rna.sysu.edu.cn/rmbase/) [28], multiple m^6^A modification sites were found in *VPS25*, and YTHDC1 is an RNA-binding protein (RBP) of *VPS25*. Therefore, we detected the m^6^A modification of *VPS25* in glioma cells by m^6^A RIP-qPCR analysis. Compared with the IgG control, the m^6^A antibody levels of VPS25 in U87MG and U251 cells were increased 22- and 25-fold, respectively (Fig. 7A).

METTL3 and METTL14 are essential m^6^A methyltransferases and have been reported to be involved in tumorigenesis of glioblastoma stem cells [29]. YTHDC1 is an m^6^A-binding nuclear protein with a YTH domain [30]. To explore the possible mechanisms of m^6^A-regulated expression of *VPS25*, *METTL3*, *METTL14*, and *YTHDC1* were knocked down in glioma cells. RT-qPCR assays in U87MG and U251 verified the KD efficiency (Additional file 11: Fig. S9A–C). The m^6^A quantitative analysis showed that the *METTL3* and *METTL14* KD decreased the total m^6^A modification level in glioma cells, whereas the *YTHDC1* KD did not (Additional file 11: Fig. S9D). The mRNA level of *VPS25* was upregulated in glioma cells with KD of *METTL3* or *METTL14*, but it was not affected in the *YTHDC1* KD cells (Fig. 7B–D). However, the protein level of VPS25 was not affected in glioma cells with KD of *METTL3* or *METTL14*, but it was upregulated in the *YTHDC1* KD cells (Fig. 7E). These data suggest that METTL3 or METTL14-mediated m^6^A is associated with the downregulation of *VPS25* mRNA in glioma. In turn, YTHDC1 may regulate the protein expression of VPS25.

We then speculated that METTL3, METTL14, and YTHDC1 might affect the proliferation of glioma cells through VPS25. We decreased *VPS25* expression in *METTL3*, *METTL14*, and *YTHDC1* KD glioma cells, and a series of restoration assays were performed. KD of *METTL3* and *METTL14* inhibited cell proliferation, whereas KD of *YTHDC1* promoted cell proliferation. Silencing *VPS25* decreased the proliferation in *YTHDC1*-KD U251 cells, but not in *METTL3* and *METTL14*-KD cells (Fig. 7F and Additional file 11: Fig. S9E and F). Together, these results indicate that YTHDC1 may inhibit glioma proliferation by reducing VPS25 expression.

## Discussion

ESCRT acts as a complex primarily to sort ubiquitinated receptors into MVBs, which are then degraded by the lysosomes. During this process, each ESCRT subunit performs its functions concomitantly on membrane bending and scission [31]. In this study, we screened the expression of ESCRT subunits in glioma from TCGA and GEO databases. However, the expression trends of different ESCRT subunits are inconsistent. For instance, *MVB12A*, *VPS25*, *CHMP2A*, and *IST1* were significantly upregulated in glioma, whereas *VPS36* and *CHMP1B* were significantly downregulated (Table 1). As reported previously, ESCRT components have been implicated in both oncogenic and tumor suppressor function in various types of human cancer [17, 18]. Interestingly, VPS25 and VPS36, which belong to ESCRT-II, have opposite trends. VPS36 are under-expressed in advanced prostate cancer and have been associated with prostate cancer cell proliferation [32]. Therefore, we speculate that VPS25 may function independently of the ESCRT complex in glioma.

The ESCRT complex has been confirmed to participate in several key biological functions of cancer development, including proliferation, cell cycle, invasion, autophagy, and exosomes [8]. However, the function of VPS25 in tumors remains largely unknown. Here, we determined that the *VPS25* deficiency significantly inhibited glioma cell proliferation by inducing the G0/G1 phase arrest of the cell cycle (Figs. 2 and 3). By western blot, we found that VPS25 regulates the protein expression of CDK2, cyclin E, and p21 (Fig. 6). Cyclin E binds and activates CDK2, and thus promotes transition through G1/S and G2/M phases. Meanwhile, p21 encodes a cyclin-dependent kinase inhibitor, which binds to and inhibits the activity of cyclin E-CDK2 complexes. Therefore, p21 mediates cell cycle arrest at the G1/S and G2/M phases [33]. Previous studies have shown that the RAS association domain family 1 isoform A (RASSF1A) induces cell cycle arrest in the G1 phase with observed reduced expression of cyclin E and increased levels of p21 [34]. In addition, PKD1 expression induces cell cycle arrest in G0/G1 and upregulates p21 [35]. As a component of the ESCRT-1 complex, TSG101 deficiency in cancer cells was accompanied by growth inhibition, cell cycle arrest, apoptosis, and an increased abundance of p21 mRNA and protein [36]. These data strongly suggest that VPS25 may regulate its effects on the cell cycle and proliferation of glioma cells mainly by regulating cyclin E, CDK2, and p21 expression.

We further investigated the mechanism by which VPS25 promotes glioma progression. JAK (janus kinase)-STAT (signal transducer and activator of transcription) signaling pathway is crucial in various physiological processes, including immune function, cell growth, differentiation, and hematopoiesis [37]. Studies have shown that its deregulation contributes to the emergence and progression of various cancers, including glioma [38]. Previous research showed that inhibition of the JAK-STAT signaling pathway blocked the cell cycle at the G0/G1 phase [39]. Moreover, dysregulation of the JAK-STAT signaling pathway in *Drosophila* VPS25 mutant tissues is associated with tumorigenesis [40]. In this study, the phosphorylation of JAK-STAT was downregulated by *VPS25* KD, whereas it was upregulated by *VPS25* overexpression. This finding indicates that VPS25 may regulate glioma growth through the JAK-STAT pathway.

*N*^6^-methyladenosine (m^6^A) is the most abundant internal modification in eukaryotic mRNAs [41]. The METTL3 and METTL14, which are the methyltransferase complex's main components, catalyzed the formation of m^6^A modification [42]. YTHDC1 has been identified as an m^6^A direct reader that recognizes m^6^A-modified RNAs [30]. Recent research shows that KD of *METTL3* or *METTL14* promotes GSC self-renewal and tumorigenesis by upregulating the expression of oncogenes, such as *ADAM19* [43]. YTHDC1 also mediates nuclear export and splicing of *N*^6^-methyladenosine methylated mRNAs [44, 45]. We revealed that METTL3 or METTL14 only regulates the mRNA expression of *VPS25*. Furthermore, YTHDC1 only regulates VPS25 protein expression. We also demonstrated that YTHDC1 affects the proliferation of glioma cells by regulating VPS25 expression. As a nuclear m^6^A reader, YTHDC1 promotes *PTEN* mRNA degradation to increase AKT phosphorylation, thus facilitating neuronal survival after ischemia [46]. This molecular mechanism should be further investigated to elucidate whether YTHDC1 affects the JAK-STAT pathway through decreases in VPS25 mRNA stability.

## Conclusions

In summary, we determined the VPS25 expression and its biological function in glioma. We found that VPS25 is overexpressed in glioma and promotes the proliferation of glioma. Additionally, VPS25 targets the p21 and JAK-STAT signaling pathways. Thus, this study not only elucidates the VPS25-dependent regulation of glioma but also provides a new putative biomarker and therapeutic target for glioma diagnosis and treatment. However, the application of VPS25 in clinical practice needs further research.

## Supplementary Information

**Additional file 1: Figure S1.** mRNA expression of all ESCRT subunits in glioma from TCGA database. *P < 0.05, **P < 0.01, ***P < 0.001. Normal: normal brain tissue; Glioma: glioma tissue.

**Additional file 2: Figure S2.** mRNA expression of all ESCRT subunits in glioma from GEO database. *P < 0.05, **P < 0.01, ***P < 0.001. Normal: normal brain tissue; Glioma: glioma tissue.

**Additional file 3: Figure S3.** Expression of ESCRT subunits in glioma tissues based on tumor grade. (A) Expression of *MVB12A*, *VPS25*, *CHMP2A*, and *IST1* in WHO grade II and WHO grade III/IV from GEO database. (B) Expression of *VPS25* in WHO grade II and WHO grade III from TCGA database. (C) Expression of *CHMP2A* in WHO grade II and WHO grade III from TCGA database. (D) The overall survival of LGG or GBM patients with high and low levels of *VPS25* was plotted from GEPIA2. (E) The correlation between VPS25 expression with IDH mutation status in glioma. *P < 0.05, ***P < 0.001.

**Additional file 4: Figure S4.** The protein expression of VPS25 in glioma. (A) The VPS25 was detected by western blot in GBM (n = 8) and normal brain tissues (n = 14). (B) Statistics of the VPS25 expression in A. N: normal brain tissues, G: GBM tissues, ***P < 0.001.

**Additional file 5: Figure S5.** The knockdown of *VPS25* in glioma cells. (A) The relative *VPS25* expression level in 2 glioma cell lines (U87MG and U251) was compared with those in 14 non tumor brain tissues. (B) The U87MG cells, which were transfected with NC and siVPS25-1, -2, -3 for 48 h, were harvested. Then RT-qPCR detected the expression of *VPS25* in U87MG cells. (C) RT-qPCR detected the expression of *VPS25* in U251 cells transfected with siRNA. Experiments were performed and presented as shown in B. ***P < 0.001. NC: negative control. siVPS25: *VPS25* gene silencer.

**Additional file 6: Figure S6.** VPS25 regulates glioma cell apoptosis. (A) TUNEL assays were performed on U87MG cells, statistical analysis is shown on the right. (B) TUNEL assays were performed on U251 cells, statistical analysis is shown on the right. Scale bars, 100 μm. Data are mean ± SD from three independent experiments. * P < 0.05, NC: negative control. siVPS25: *VPS25* gene silencer.

**Additional file 7: Table S1.** The mRNAs expression between NC and siVPS25.

**Additional file 8: Table S2.** Top KEGG enrichment for the down-expressed mRNAs.

**Additional file 9: Figure S7.** The p-JAK2,3 and p-STAT2,3 was detected by western blot in NC and siVPS25 glioma cells.

**Additional file 10: Figure S8.** The function of siVPS25-1 in glioma cells. (A) RTCA xCELLigence assay showed that transfection of siVPS25-1 suppressed the proliferation of U251 cells. (B) Transfection of siVPS25-1 suppressed the colony formation of U251 cells. (C) The *VPS25*-silenced U251 cells were arrested at the G0/G1 phase of the cell cycle. (D) The p-JAK1 and p-STAT1 was detected by western blot in NC and siVPS25-1 glioma cells. Data are mean ± SD from three independent experiments. *P < 0.05, **P < 0.01, ***P < 0.001. NC: negative control. siVPS25: *VPS25* gene silencer.

**Additional file 11: Figure S9.** The knockdown of *METTL3*, *METTL14*, and *YTHDC1* in glioma cells. (A) RT-qPCR was performed to verify the knockdown efficiency of *METTL3* in U87MG and U251 cells. (B) RT-qPCR was performed to verify the knockdown efficiency of *METTL14* in U87MG and U251 cells. (C) RT-qPCR was performed to verify the knockdown efficiency of *YTHDC1* in U87MG and U251 cells. (D) The total m^6^A modification level was detected in *METTL3*, *METTL14*, and *YTHDC1*-knockdown cells. (E) U251 glioma cells were used to evaluate proliferation after being transfected with siVPS25 and siMETTL3. (F) U251 glioma cells were used to evaluate proliferation after being transfected with siVPS25 and siMETTL14. *P < 0.05, **P < 0.01, ***P < 0.001. ns: no significant.

## Data Availability

The data used to support the findings of this study are included within the article.

## Abbreviations

CNS: Central nervous system
WHO: World Health Organization
NCCR: National Central Cancer Registry of China
ESCRT: Endosomal sorting complex required for transport
ILVs: Intraluminal vesicles
MVBs: Multivesicular bodies
TCGA: The Cancer Genome Atlas
GEO: Gene Expression Omnibus
NC: Negative control
KD: Knockdown
KEGG: Kyoto encyclopedia of genes and genomes
m^6^A: *N*^6^-Methyladenosine
RBP: RNA-binding protein
RASSF1A: RAS association domain family 1 isoform A
JAK: Janus kinase
STAT: Signal transducer and activator of transcription
GEPIA2: Gene Expression Profiling Interactive Analysis 2
GTEx: Genotype-Tissue Expression
ATCC: American Type Culture Collection
DMEM: Dulbecco's modified Eagle medium
FBS: Fetal bovine serum
PI: Propidium iodide
DEGs: Differentially expressed genes
ECL: Enhanced chemiluminescence
RIP: RNA immunoprecipitation
GBM: Glioblastoma multiforme
LGG: Lower grade glioma
