# Supplementary figures and images for: YTHDC1-mediated VPS25 regulates cell cycle by targeting JAK-STAT signaling in human glioma cells
Source: Cancer Cell Int. 2021 Dec 4;21:645. doi: 10.1186/s12935-021-02304-0 (PMC8642909; doi:10.1186/s12935-021-02304-0)

Figure S1

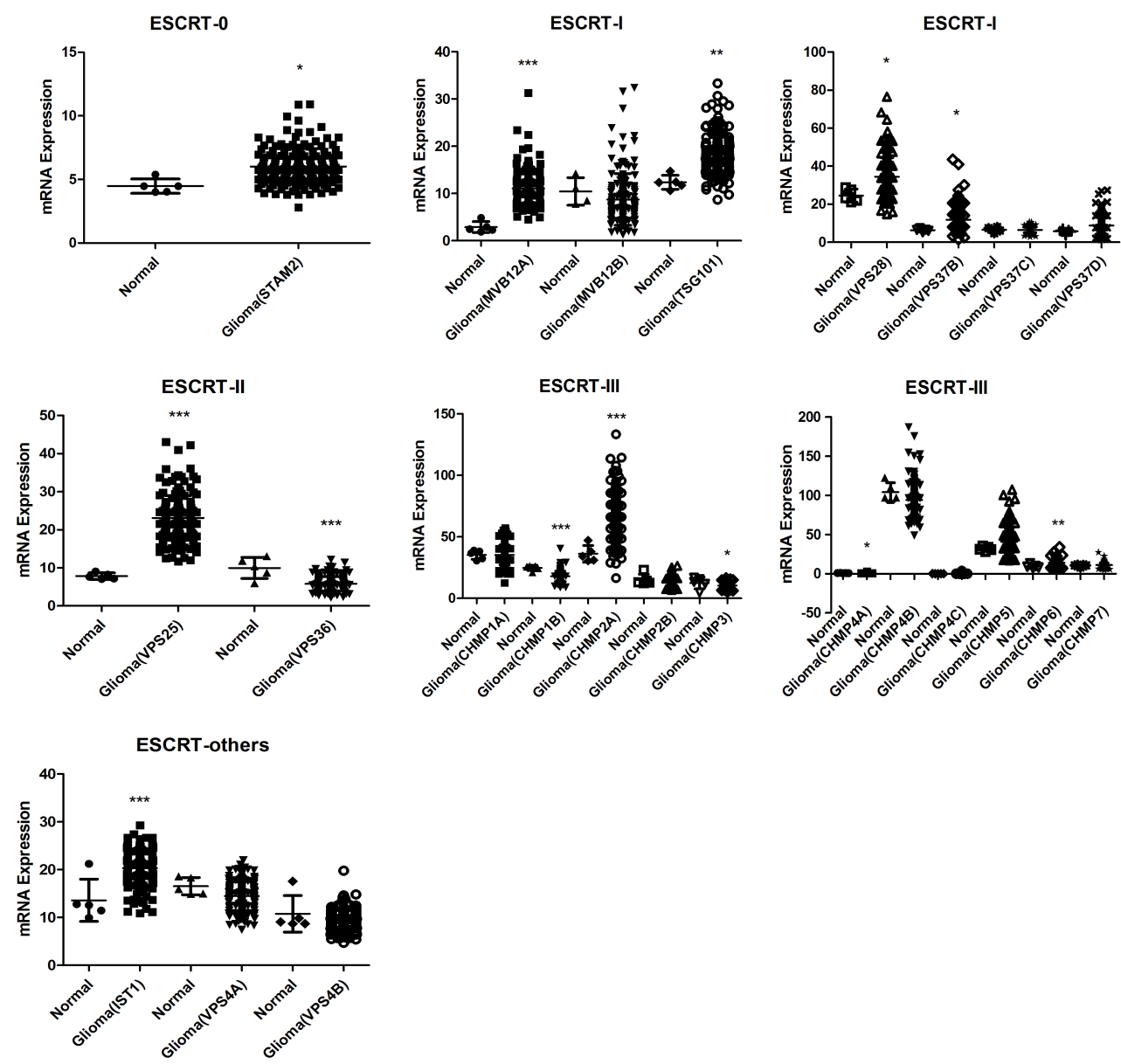

Supplement: Supplementary file 1 — Additional file 1: Figure S1. mRNA expression of all ESCRT subunits in glioma from TCGA database. *P < 0.05, **P < 0.01, ***P < 0.001. Normal: normal brain tissue; Glioma: glioma tissue. [file 12935_2021_2304_MOESM1_ESM.pdf]

Figure S2

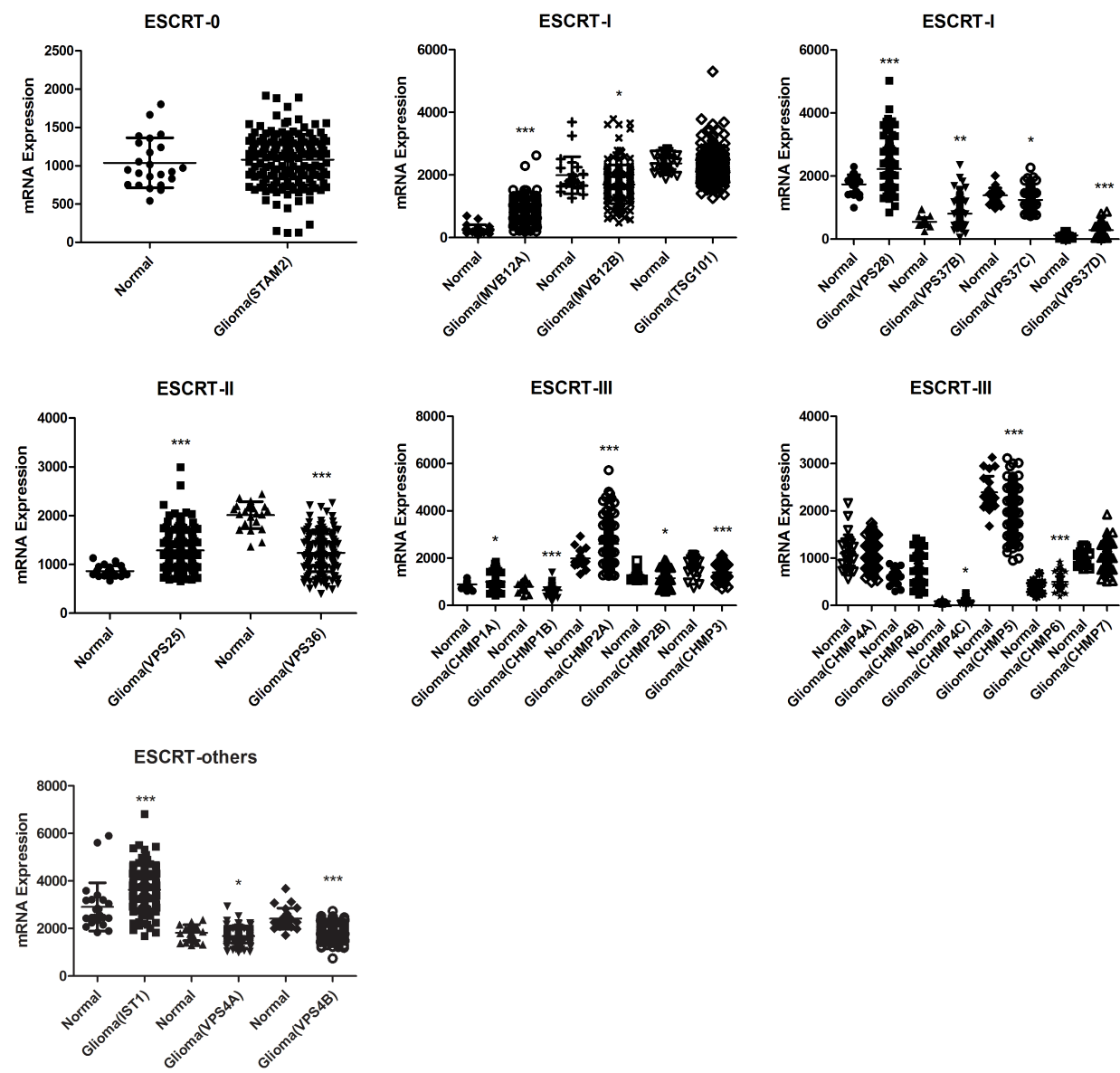

Supplement: Supplementary file 2 — Additional file 2: Figure S2. mRNA expression of all ESCRT subunits in glioma from GEO database. *P < 0.05, **P < 0.01, ***P < 0.001. Normal: normal brain tissue; Glioma: glioma tissue. [file 12935_2021_2304_MOESM2_ESM.pdf]

Figure S3

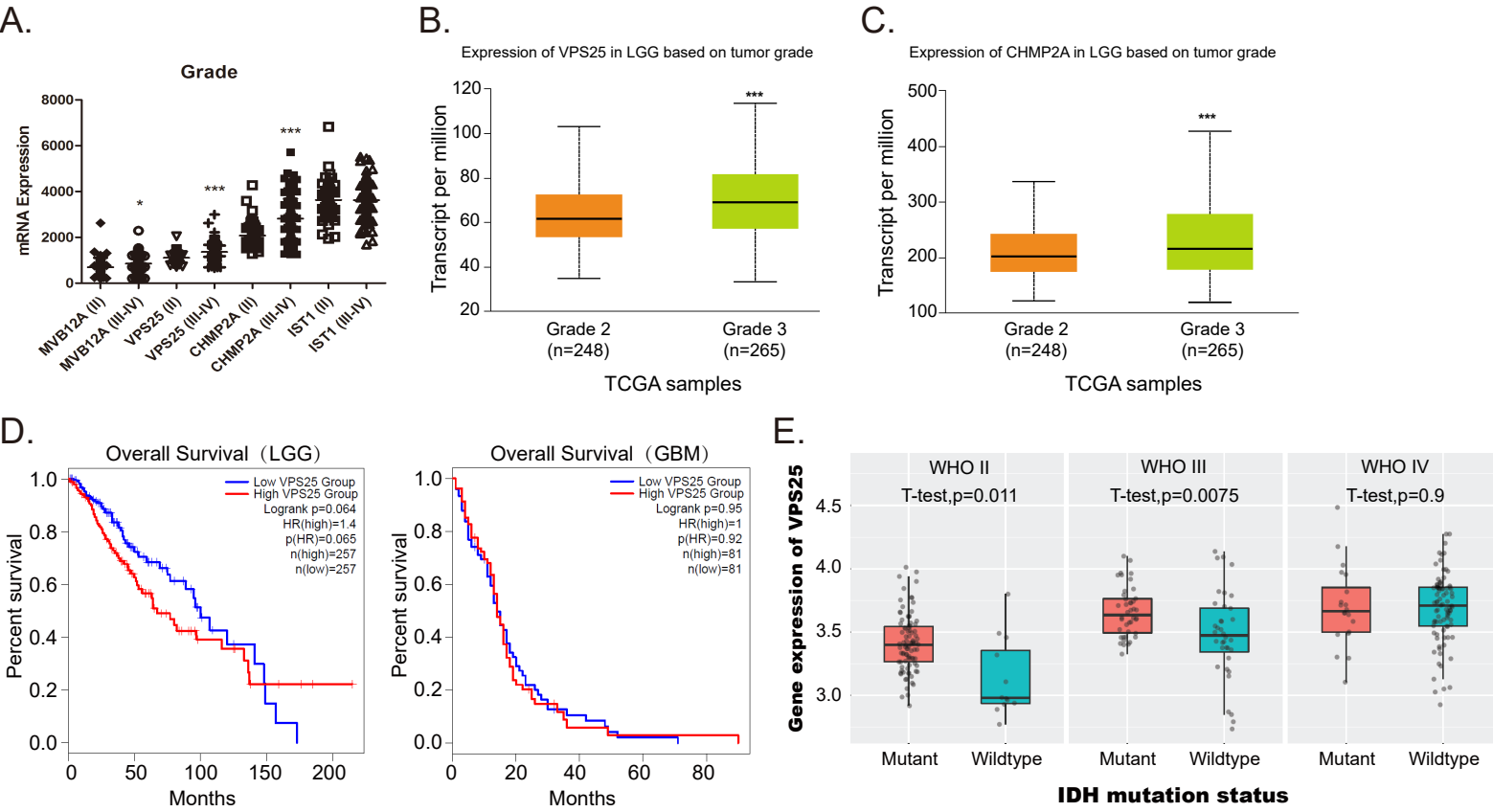

Supplement: Supplementary file 3 — Additional file 3: Figure S3. Expression of ESCRT subunits in glioma tissues based on tumor grade. (A) Expression of MVB12A, VPS25, CHMP2A, and IST1 in WHO grade II and WHO grade III/IV from GEO database. (B) Expression of VPS25 in WHO grade II and WHO grade III from TCGA database. (C) Expression of CHMP2A in WHO grade II and WHO grade III from TCGA database. (D) The overall survival of LGG or GBM patients with high and low levels of VPS25 was plotted from GEPIA2. (E) The correlation between VPS25 expression with IDH mutation status in glioma. *P < 0.05, ***P < 0.001. [file 12935_2021_2304_MOESM3_ESM.pdf]

Figure S4

A.

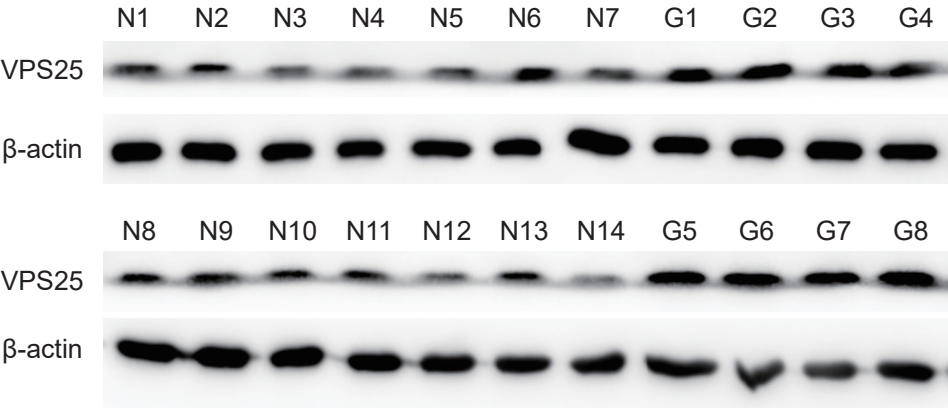

B.

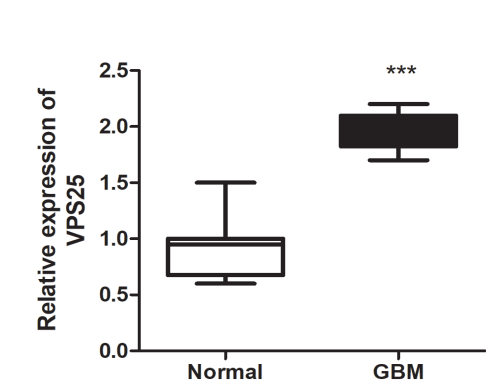

Supplement: Supplementary file 4 — Additional file 4: Figure S4. The protein expression of VPS25 in glioma. (A) The VPS25 was detected by western blot in GBM (n = 8) and normal brain tissues (n = 14). (B) Statistics of the VPS25 expression in A. N: normal brain tissues, G: GBM tissues, ***P < 0.001. [file 12935_2021_2304_MOESM4_ESM.pdf]

Figure S5

A.

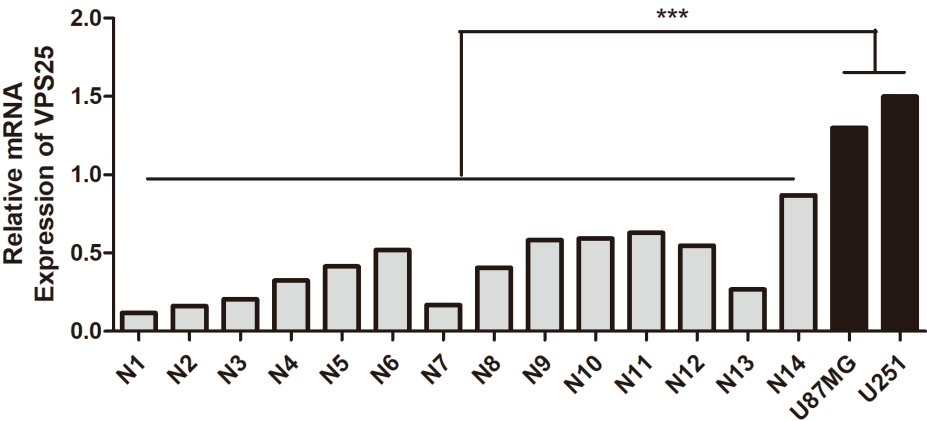

B.

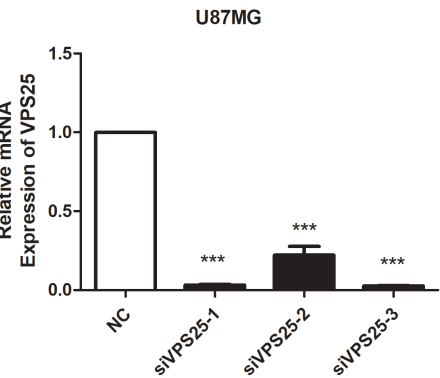

C.

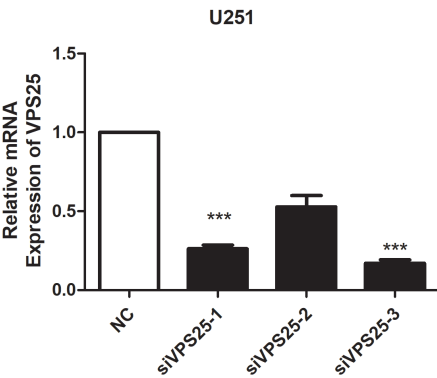

Supplement: Supplementary file 5 — Additional file 5: Figure S5. The knockdown of VPS25 in glioma cells. (A) The relative VPS25 expression level in 2 glioma cell lines (U87MG and U251) was compared with those in 14 non tumor brain tissues. (B) The U87MG cells, which were transfected with NC and siVPS25-1, -2, -3 for 48 h, were harvested. Then RT-qPCR detected the expression of VPS25 in U87MG cells. (C) RT-qPCR detected the expression of VPS25 in U251 cells transfected with siRNA. Experiments were performed and presented as shown in B. ***P < 0.001. NC: negative control. siVPS25: VPS25 gene silencer. [file 12935_2021_2304_MOESM5_ESM.pdf]

Figure S6

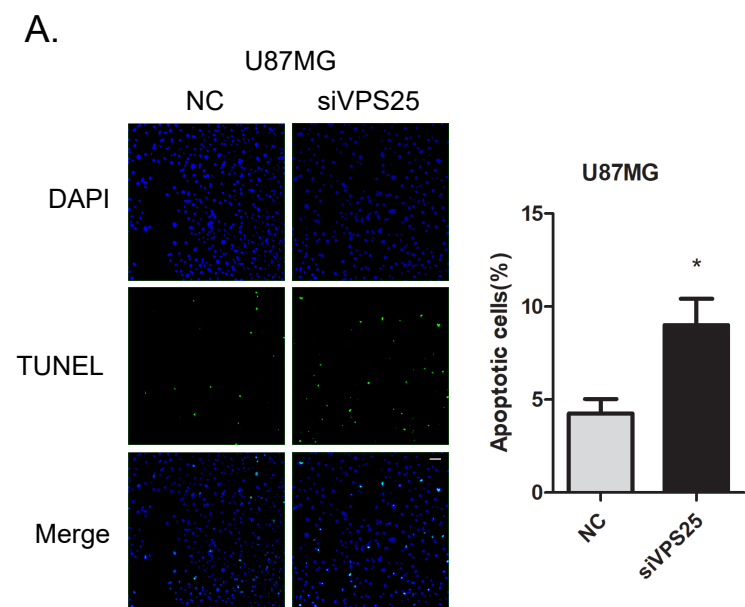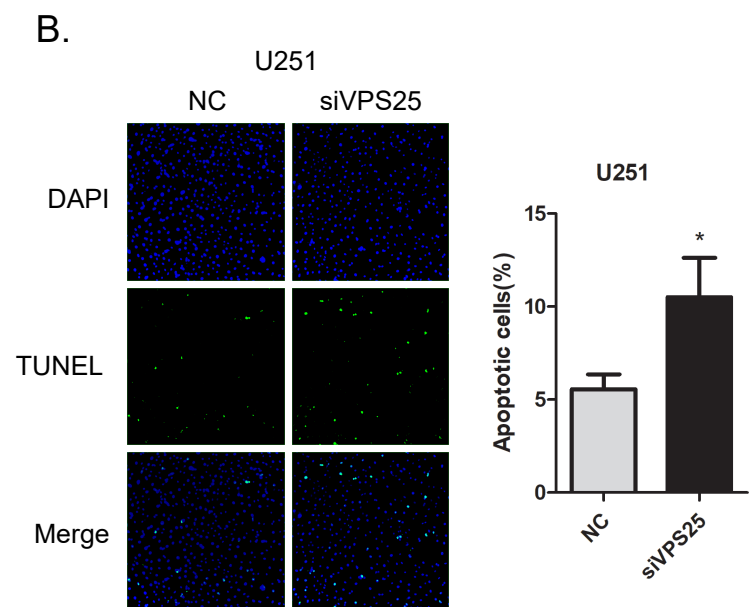

Supplement: Supplementary file 6 — Additional file 6: Figure S6. VPS25 regulates glioma cell apoptosis. (A) TUNEL assays were performed on U87MG cells, statistical analysis is shown on the right. (B) TUNEL assays were performed on U251 cells, statistical analysis is shown on the right. Scale bars, 100 μm. Data are mean ± SD from three independent experiments. * P < 0.05, NC: negative control. siVPS25: VPS25 gene silencer. [file 12935_2021_2304_MOESM6_ESM.pdf]

Figure S7

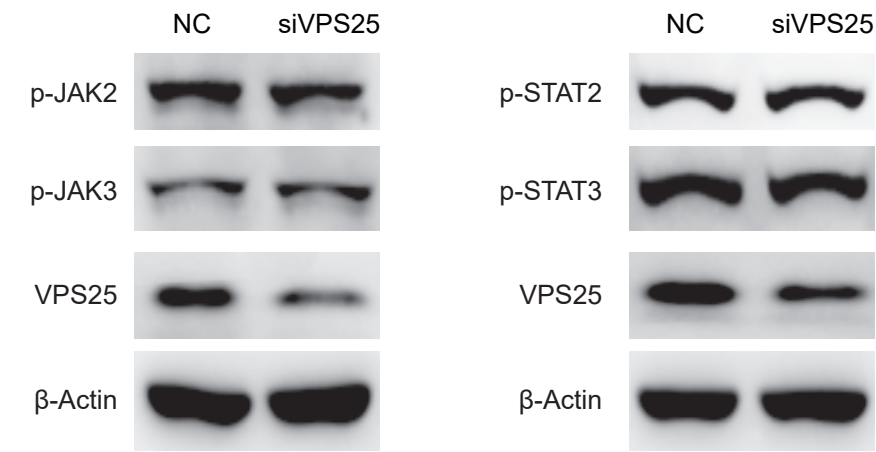

Supplement: Supplementary file 9 — Additional file 9: Figure S7. The p-JAK2,3 and p-STAT2,3 was detected by western blot in NC and siVPS25 glioma cells. [file 12935_2021_2304_MOESM9_ESM.pdf]

Figure S8

A.

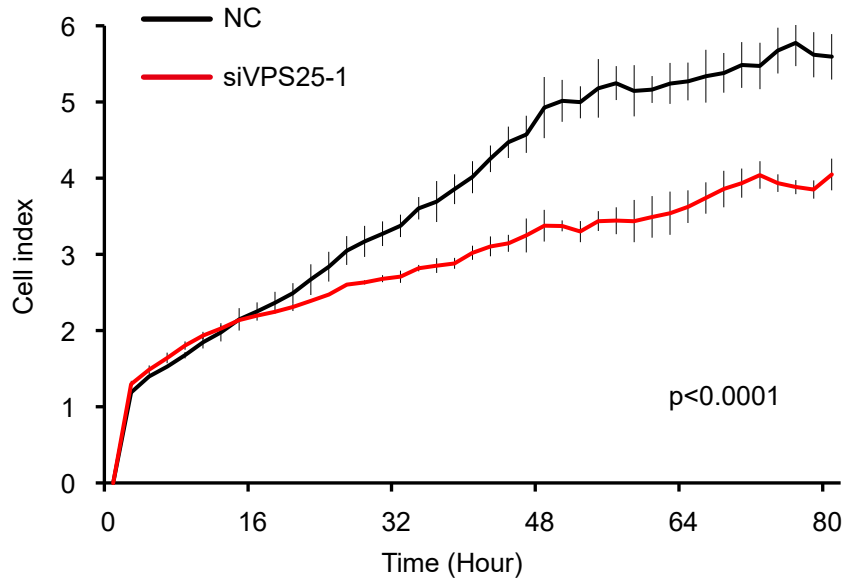

B.

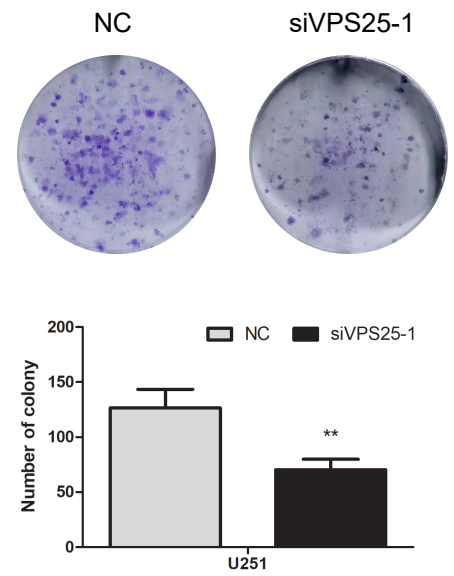

C.

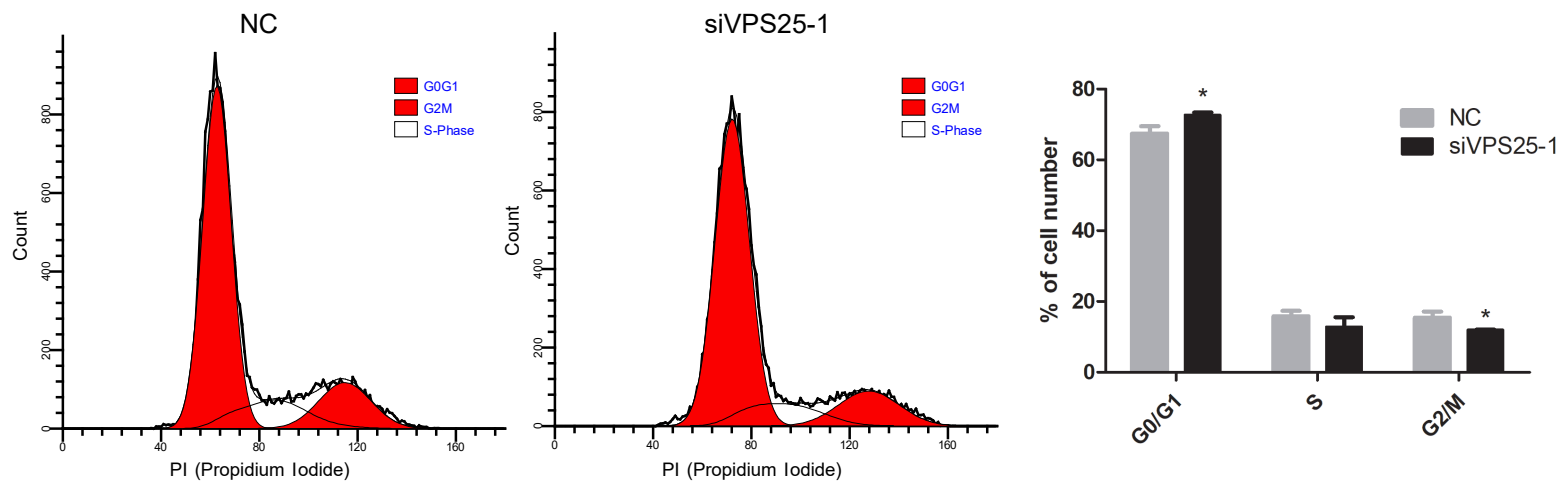

D.

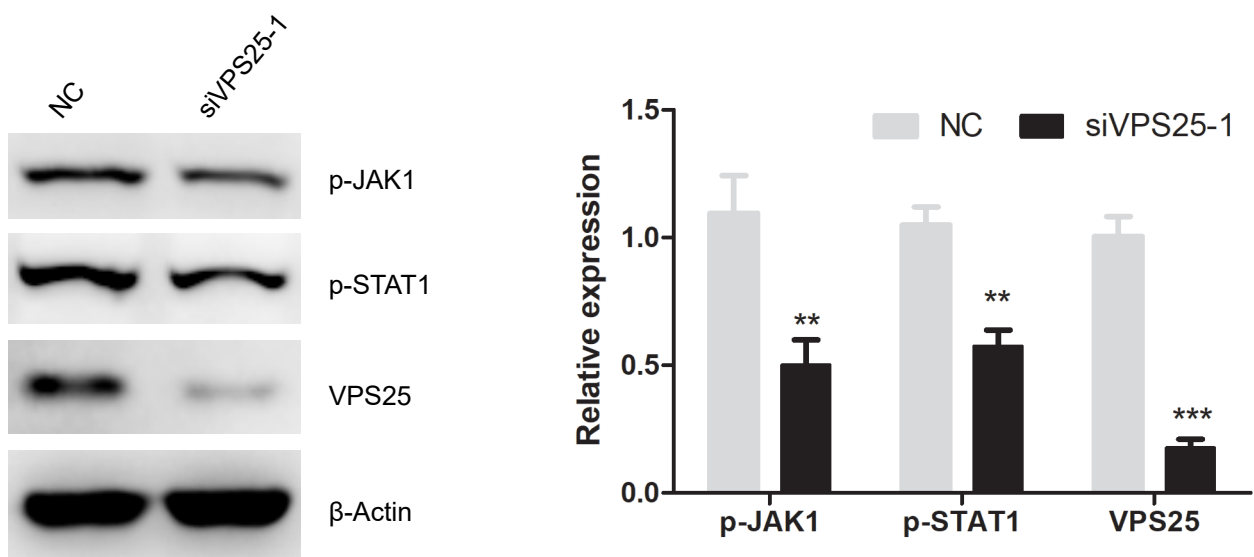

Supplement: Supplementary file 10 — Additional file 10: Figure S8. The function of siVPS25-1 in glioma cells. (A) RTCA xCELLigence assay showed that transfection of siVPS25-1 suppressed the proliferation of U251 cells. (B) Transfection of siVPS25-1 suppressed the colony formation of U251 cells. (C) The VPS25-silenced U251 cells were arrested at the G0/G1 phase of the cell cycle. (D) The p-JAK1 and p-STAT1 was detected by western blot in NC and siVPS25-1 glioma cells. Data are mean ± SD from three independent experiments. *P < 0.05, **P < 0.01, ***P < 0.001. NC: negative control. siVPS25: VPS25 gene silencer. [file 12935_2021_2304_MOESM10_ESM.pdf]

Figure S9

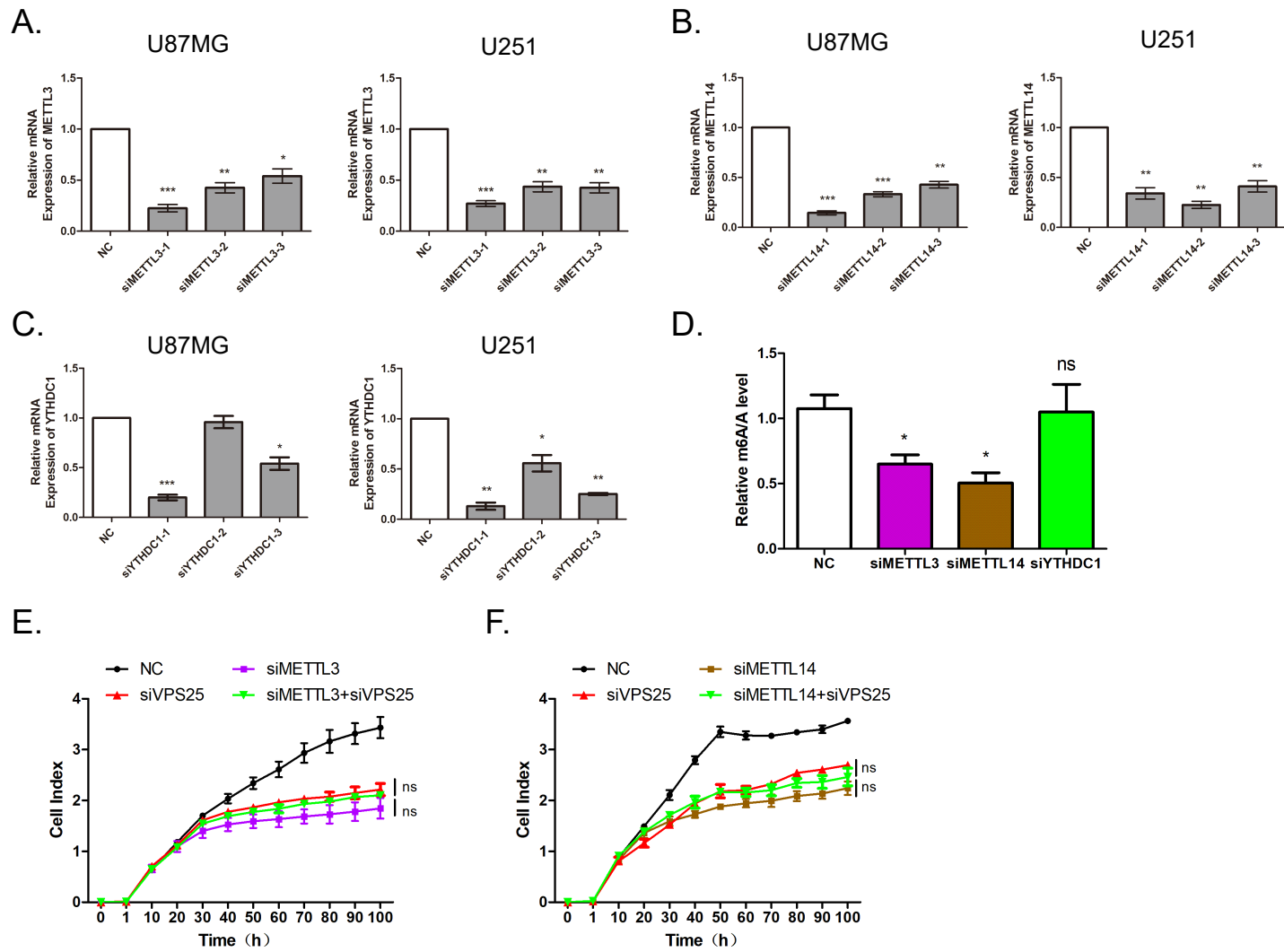

Supplement: Supplementary file 11 — Additional file 11: Figure S9. The knockdown of METTL3, METTL14, and YTHDC1 in glioma cells. (A) RT-qPCR was performed to verify the knockdown efficiency of METTL3 in U87MG and U251 cells. (B) RT-qPCR was performed to verify the knockdown efficiency of METTL14 in U87MG and U251 cells. (C) RT-qPCR was performed to verify the knockdown efficiency of YTHDC1 in U87MG and U251 cells. (D) The total m6A modification level was detected in METTL3, METTL14, and YTHDC1-knockdown cells. (E) U251 glioma cells were used to evaluate proliferation after being transfected with siVPS25 and siMETTL3. (F) U251 glioma cells were used to evaluate proliferation after being transfected with siVPS25 and siMETTL14. *P < 0.05, **P < 0.01, ***P < 0.001. ns: no significant. [file 12935_2021_2304_MOESM11_ESM.pdf]
